# Investigation on pollination approaches, reproductive biology and essential oil variation during floral development in German chamomile (*Matricaria chamomilla* L.)

**DOI:** 10.1038/s41598-022-19628-0

**Published:** 2022-09-10

**Authors:** Niketa Yadav, Pooja Shakya, Ajay Kumar, Rahul Dev Gautam, Ramesh Chauhan, Dinesh Kumar, Ashok Kumar, Sanatsujat Singh, Satbeer Singh

**Affiliations:** 1 Division of Agrotechnology, Council of Scientific and Industrial Research - Institute of Himalayan Bioresource Technology, Palampur, Himachal Pradesh 176 061 India; 2 Academy of Scientific and Industrial Research (AcSIR), Ghaziabad, 201002 India; 3 Division of Chemical Technology, Council of Scientific and Industrial Research - Institute of Himalayan Bioresource Technology, Palampur, Himachal Pradesh 176 061 India

**Keywords:** Plant breeding, Flowering

## Abstract

German chamomile is an important medicinal and aromatic herb known for its blue essential oil. It lacks studies on anthesis, breeding systems and floral development with their impact on the essential oil. Therefore, the study investigated floral development and divided it into six reproductive stages (RS-1 to RS-6). The first four stages (5–6 days long) were identified as the floral enlargement and differentiation, followed by the fifth stage (10 days long) of three anthesis flushes, *i.e.,* anther dehiscence, ray and disc florets' style branches flush. Anther dehiscence started 1–2 days before style branches flushes showed protandry and overlapped later with style branches flushes. Pollen production started from RS-3 and showed maximum viability (89%) at anther dehiscence (RS-5.1). Pollen showed dispersal through the air up to 0.7 m distance. Seed setting in controlled pollination experiments showed that removing disc florets could be successfully used as the emasculation alternate in German chamomile. The maximum essential oil content (0.40%) at the full blossomed floral stage (RS-4 &-5) suggested the right time for capitula harvesting. The findings on reproductive biology and breeding systems would offer several tools and techniques to support future breeding programs for genetic improvement of German chamomile.

## Introduction

German chamomile (*Matricaria chamomilla* L.) is an annual herb abundantly found in southeast Europe and adjoining Asian countries. It can be grown on a wide range of soil types and tolerate cold and soil alkalinity. This species has solitary, pedunculated, terminal, radiate and heterogamous capitula with pistillate fertile ray florets and, monoclinous fertile disc florets. Due to its extensive aromatic and pharmaceutical properties, chamomile is known as the "star" of medicinal and aromatic plants^1^. The part of the plant that has an economic interest is the dry capitula, its extracts, and blue essential oil. Chamomile capitula extracts have been extensively used in herbal remedies for thousands of years^2^. It is used in several industrial products like herbal tea, cosmetics, food flavors, dye and pest repellent^3^. In addition to pharmaceutical uses, the blue essential oil is extensively used in perfumery and aromatherapy industries. The blue essential oil has anti-inflammatory, antiulcerogenic, antimicrobial, antiseptic, antispasmodic, sedative, immunomodulatory and wound healing properties^4,5^, hence is being used in Arabic and homeopathic medicine formulations.

Chamazulene and α-bisabolol are the most important among the known 120 secondary metabolites in chamomile oil^6–9^. The essential oil content varies between 0.2 and 1.9 percent^10,11^, depending on the stage of capitulum development, genotype and environmental factors^12–15^. The genetic factors influencing the composition of different bisaboloids were discussed earlier by Horn et al.^16^ and Franz^17^. The α-bisabolol and chamazulene constituents are governed by a single recessive gene^18^. The evolutionary changes among the pathways mevalonate (MVA) and methylerythritol phosphate (MEP) with differentially expressed genes (DEGs), transcription factor (TF) genes, terpene synthases (TPSs)^19^ and cytochrome P450 enzymes (CYPs) may be involved at genetic level for variation in terpenoids in German chamomile^20^. Stage of pathway and level of expression of candidate genes during harvesting affect the essential oil content and composition^21,22^. Thus knowledge of the right stage of capitulum development in relation to oil content is vital.

German chamomile has great economic value and demand in the global market, even though farmers of developing countries like India are not interested in its large scale cultivation due to a lack of varieties and better agro-technologies. Thus, knowledge of reproductive biology, floral development stages, pollination behavior and breeding systems are of primary importance for researchers to decide on strategies for genetic improvement. Moreover, understanding of reproductive stages with their unique features, anthesis flushes and pollen viability will helpful in controlled pollination in several ways through identification of right stage of emasculation, way of emasculation, time of pollination and stage of pollination^23,24^. The studies on sequential happenings of anthesis flushes, breeding tools and reproductive biology are very scanty in German chamomile. However, the floral biology of a few other Asteraceae family members is reported earlier^25,26^. In this context, the objectives of the present study are twofold: (1) to identify floral developmental stages and to describe the reproductive biology and chamomile breeding system based on key characteristics like size of florets, phases of anthesis, length of the pistil, pollen viability, and plant growth stages and (2) to study essential oil content and composition to determine the optimum harvest index for maximum oil content and quality.

## Materials and methods

### Plant material and study site

A diploid population "IHMC203P" of Indian origin German chamomile was grown during the winter season (November to April 2020) at the experimental farm of Agrotechnology Division, CSIR Institute of Himalayan Bioresource Technology, Palampur, Himachal Pradesh (India). The experimental site is at 32°6′25" N, 76°33′30" E, and 1300 m above mean sea level. The location comes under the mid-hill zone (Zone-II agro-climatological), having a humid sub-temperate climate and high mean annual rainfall (∼2500 mm). During the crop season (November to April 2020), the total rainfall was 802 mm, the average weekly temperature ranged between 2 and 34 °C, and relative humidity ranged from 46 to 81%. The soil of the experimental site was acidic-silty clay loam with pH ranging from 5.23 to 6.10 and having 1.40 to 2.01% available carbon. The pH, electric conductivity and total dissolved solids of irrigation water were varied from 6.01 to 6.71, 0.06 to 0.12 mS/cm and 92 to 312 ppm, respectively at 25℃. Floral characterization, floral development, and chemical characterization were studied in the main plot grown under open field conditions. To check breeding systems, plants had grown in pots under rainout shelter. A total of three manual weedings and nine flood irrigations were applied as per the requirement for a good crop growth in open filed conditions. A dose of 15 t ha^−1^ of well-decomposed farmyard manure was given before field preparation. Further, 100:60:40 kg NPKha^−1^ given as a basal dose of 1/3^rd^ N plus full P and K at pre-sowing, and the remaining N was given at vegetative growth and initiation of flowering in equal amounts. The sampling, design and replications details are given in their respective headings.

### Floral characterization and development

Three random plants representing the three replications for the floral characterization, anthesis and pollen studies were labeled in the main plot. Further, three flower heads from each pre-selected plant were observed daily in the forenoon to study types and length of florets; length of style and stamen; anthesis flush, pattern of anthesis and floral development stages, independently. The capitulum developmental process was observed from primordium initiation to distinguish floral differentiation. A light microscope with Magnus CMOS10 MP C-Mount camera and Nikon –DSLR camera were used to study different floral development stages and capture photographs. The length of florets, styles and stamens were measured on glass slides with 1/10 µm scales under a light microscope. The complete developmental process was partitioned into distinct stages with key characteristics like floral primordia initiation, further increase in size, developments in stamen and style, opening of florets, pollen viability, senescence of style and seed formation stages. Data on floret length, pistil length and capitulum diameter were recorded at various stages to define full floral developmental events.

### Anthesis flushes, pollen viability and dispersal

Five random floral primordia from each of the three pre-selected plants were labeled to study anthesis time and duration. The capitula were observed daily up to the end of anthesis in each floret. The time and duration of the florets' emerging style branches and stamens were recorded. Pollen viability was examined at different floret developmental stages and intervals after anther dehiscence. The anthers were carefully dissected from disc florets, then crushed on a glass slide and stained with acetocarmine. Also, the anthers of matured florets at the time of dehiscence were crushed on five different glass slides, of which individual slides were stained with acetocarmine at a gap of every two hours up to 10 h. The stained slides were observed under a light microscope at 40X and analyzed to assess pollen viability. The experiment on pollen dispersal was conducted in the open field. For that, glycerin wet glass slides were kept in all four directions around chamomile plots for one hour from 10:00 to 11:00 AM on a sunny day. The glass slides were repeated at every 10 cm gap away from the plants in the first meter and at every 50 cm gap up to the following 5 m.

### Experiments on breeding systems and seed set

Three controlled pollination experiments viz., self-pollination, manual cross-pollination and open pollination were performed to study breeding systems in chamomile. For that, 30 plants were maintained in pots of size 20 cm under a rainout shelter to avoid the damage of bagged flower heads during rains, which is open from all sides. All recommended agronomic practices were followed for a good plant growth in the pots under rainout shelter. More than 25 flower heads were pollinated in triplicate for each of the three pollination experiments. In the first selfing experiment, a complete floral primordium remained bagged till maturity. While in the second situation, selfed floral primordium was opened one to two days before anthesis, and the disc florets were removed carefully with the help of forceps. Further, on the day of anthesis or one to two days after emasculation, the ray florets were pollinated with pollen from other pre-bagged flower heads. The crossing was done as head-to-head contact by detaching the pollinator capitulum from its mother plant. For the third experiment of open pollination, undisturbed open capitula were taken at maturity. The bags were removed at the pre-maturity stage by considering the condition of the open-pollinated capitula. A set of seeds from the above three experiments was examined for viability by germination test. For that, 100 seeds from each self, cross and open pollination experiments were taken and sown on germination paper in three petri plates, ensuring proper moisture at room temperature. Seed germination was recorded for up to 3 weeks. Seed setting percentage in all controlled pollination experiments was calculated as the number of seeds germinated to the total number of florets multiplied by a hundred.

### Essential oil extraction and characterization

The essential oil was extracted from fresh capitula collected from three different stages, i.e., pre-blossom (before anthesis), full blossom (during anthesis) and end of blossom (post anthesis). For that, the subplots of 1 m × 1 m size were marked within the main plot by avoiding the border plants. The subplots were harvested in three replications for each treatment (pre-blossom, full blossom and end of blossom). Care has been taken during capitulum picking so that the capitula other than the targeted stage should not be picked. The variances were partitioned using analysis of variance (ANOVA) of a completely randomized design.

### Ethical approval

Experimental research and field studies on plants comply with relevant institutional, national, and international guidelines and legislation.

#### Hydrodistillation by Clevenger

The essential oils were obtained immediately after capitula harvest to avoid any changes in the quantity and quality of essential oil ^27,28^, and content in percent was calculated based on the oven-dried mass remained in the flask. The essential oils were obtained by hydrodistillation in a Clevenger apparatus under optimal operating conditions. About fifty-gram capitula from each of three different stages were added to 1000 ml of distilled water in a 2-L round bottom flask. The set was placed in a heating mantle attached to tap water to ensure condensation of essential oils for 6 h^29^. Each sample afforded a blue-coloured organic phase (essential oil) with a characteristic fruity fragrance aqueous phase (aromatic water) denser than oil. Experiments were conducted thrice for each condition. The oil separated was dissolved in dichloromethane (10 ml), filtered, dried over anhydrous sodium sulfate, and used for GC–MS analysis.

#### Gas chromatography-mass spectrometry (GC–MS) analysis

The concentrated extracts were further analyzed on a Schimadzu GCMS-QP2010 (Shimadzu Corporation, Japan) system^30^. An SH-RX-5Si/MS (30 m × 0.25 mm × 0.25 µm film thickness) column fused with silica capillary was used with nitrogen as a carrier gas at a flow rate of 1 ml/min. The Injector temperature was set to 250 ℃, and the sample injection volume was 2 µl. The Oven temperature was programmed to hold at 70 ℃ for 3.0 min and then increase to 4 ℃ to 220 ℃ for 5.0 min, respectively. The ion source temperature was 200 ℃, and the interface temperature was 250 ℃. Electron energy was 0.85 eV. The compounds' retention indices (RI) relative to a mixture of n-Alkanes (C_9_–C_24_) were determined. Further, compounds were characterized by comparing their spectra with those available from MS libraries such as the National Institute of Standards and Technology (NIST) database and identifying essential oil components by Gas chromatography/Mass spectrometry^31^.

### Statistical analysis

Mean values of pistil length, stamen length, floret length, pollen viability, seed setting and essential oil content over the triplicate experiments were used to create bar diagrams and other graphical representations in Microsoft Excel 2016. Analysis of variance for all the observed parameters was carried out as per one-factor F-statistics in a completely randomized design using OPSTAT^32^. Multivariate principal component analysis was conducted to determine the synteny of different essential oil constituents towards the variation over floral developmental stages using PAST 3 software version 4.05^33^.

## Results and discussion

### Phenology of plant growth

**Figure 1:**
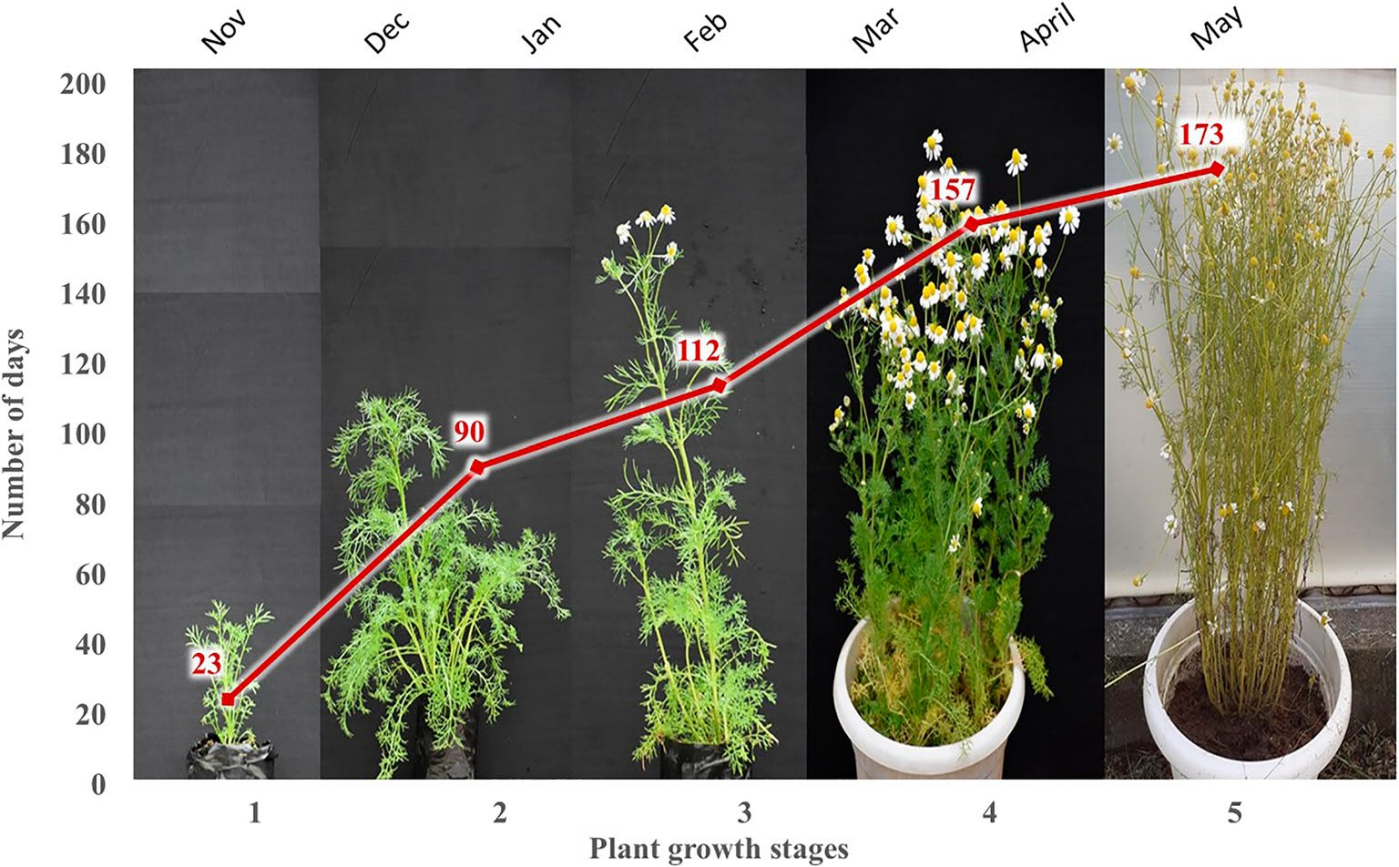
Phenological growth stages (1) seedling establishment; (2) vegetative growth; (3) initiation of flowering; (4) full flowering flush, and (5) maturity of German chamomile in Himalayan low hill conditions.

The total life span of chamomile could be divided into five stages: seedling establishment, vegetative growth, initiation of flowering, full flowering flush, and maturity (Fig. 1). Seedlings of chamomile were established in two to three weeks, followed by almost three months of vegetative growth. Flowering was initiated 112 days after sowing in February in Himalayan low hill conditions. The capitulum harvest stage was attained from 131 to 157 days during the full flowering flush stage, and seed maturity took 173 days. The initially slow growth at the seedling establishment and early vegetative growth due to lower temperature during December-January resulted in the extended crop period in the low hills of the Himalayan region^34^. The crop duration in main commercial plots can be reduced by raising nurseries and transplanting 3–4 week old seedlings^35^.

### Floral characterization

German chamomile capitula have 16 to 24 ray florets with the true ray corollas. At maturity, each ray floret develops dark brown cypselae. Disc florets vary from 182 to 330 in numbers per capitulum, have yellow tubular corollas, and anthers are linear and bi-lobed. The florets' extremely small length (2–3 mm) makes hand emasculation impossible in chamomile. The availability of polygamous conditions (separate pistillate ray florets and monoclinous disk florets) may facilitate plant breeders to perform controlled crossing by practicing emasculation via removing disc florets. The capitulum characters of this species resembled the typical characters of the Asteraceae family reported earlier^18,19^ in other species. The polygamous condition was also available in several other crops, where it is being successfully utilized in manual crossing^36^. Likely to removing male plants in papaya 37, removing male inflorescence in maize^38^ and removing staminate flowers in cucurbits etc.; removing staminate disc florets in this plant might be the tool for manual crossing. Also, this finding was utilized to perform controlled crossing experiments to understand breeding systems in the present study.

### Floral developmental stages

**Figure 2:**
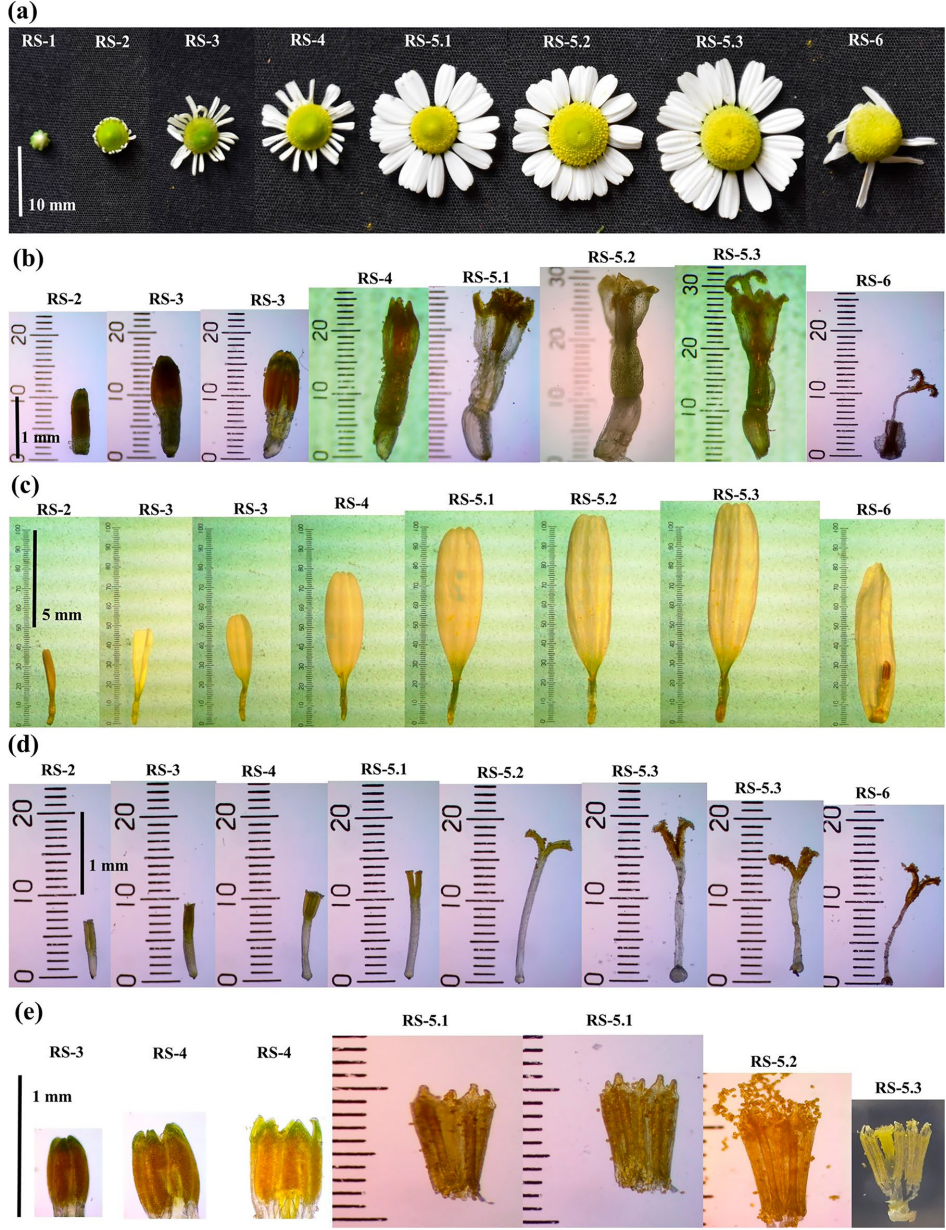
Floral development in German chamomile (**a**) capitulum (**b**) disc floret (**c**) ray floret (**d**) disk floret' pistil (**e**) stamen.

A single green floral primordium initiates at each branch tip, increasing in diameter to 5–7 mm and turning into yellowish-green colour in the next 4–5 days. At first, ray florets come out from involucre bracts. In the present study, floral development of chamomile was divided into six distinct stages, RS-1 (Reproductive stage-1) to RS-6 (Reproductive stage-6), identified with distinct key features *i.e.*, disc florets' pistil length (mm), stamen length (mm), disc florets' length (mm), ray florets' length (mm), capitulum diameter (mm) and pollen viability (%). Analysis of variance showed a highly significant (p ≤ 0.01) difference among all the reproductive stages for all the parameters studied (Tables 1 and 2). Similar to the present study, several other reports on aromatic crops of western Himalaya identified different reproductive stages with unique characteristics. For instance, floral development was identified in six different stages with unique characters in wild marigold^23^ and inflorescence development of palmarosa were characterized in ten reproductive stages to utilize the information in breeding programs^24^. However, reports on reproductive stages and their unique characteristics are not available in chamomile. The German chamomile reproductive stages are described with their key morphological traits (Fig. 2) as follows:

**Table 1:** Analysis of variance for all measured parameters in German chamomile.

Source of Variation	Degree of freedom	Mean squares					
		Disc florets’ pistil length	Stamen length	Disc florets’ length	Ray florets’ length	Disc diameter	Pollen viability
Reproductive stages	7	0.64**	1.66**	0.14**	28.71**	16.38**	5123.52**
Error	16	0.02	0.04	0.01	0.24	0.05	5.88
CV		9.62	8.56	11.76	5.9	3.01	6.08

Source of Variation	Degree of freedom	Seed setting	Essential oil content				
Treatments^#^	2	259.00**	0.02**				
Error	6	4.67	0.001				
CV		2.68	6.61				

**significant at *p* ≤ 0.01;

^#^different pollination approaches were the treatments for seed setting and different capitula harvesting stages were the treatments for essential oil content; CV (coefficient of variation).

**Table 2:** Mean comparisons of measured parameters during reproductive stages of German chamomile.

Reproductive stages	Disc florets’ pistil length (mm)	Stamen length (mm)	Disc florets’ length (mm)	Ray florets’ length (mm)	Disc diameter (mm)	Pollen viability (%)
RS-1	0.70 ± 0.12	1.00 ± 0.00	0.30 ± 0.01	4.10 ± 0.06	2.80 ± 0.12	0.00 ± 0.00
RS-2	0.90 ± 0.06	1.60 ± 0.12	0.60 ± 0.03	4.80 ± 0.12	5.47 ± 0.09	0.00 ± 0.00
RS-3	1.10 ± 0.01	1.80 ± 0.23	0.80 ± 0.06	5.70 ± 0.06	6.27 ± 0.13	13.00 ± 0.58
RS-4	1.40 ± 0.12	2.50 ± 0.06	0.80 ± 0.12	7.80 ± 0.00	7.00 ± 0.00	48.00 ± 1.16
RS-5.1	1.90 ± 0.00	2.70 ± 0.12	0.90 ± 0.02	10.00 ± 0.21	8.40 ± 0.15	89.00 ± 2.89
RS-5.2	2.00 ± 0.06	2.90 ± 0.06	0.90 ± 0.04	11.07 ± 0.30	9.37 ± 0.19	85.00 ± 2.31
RS-5.3	1.60 ± 0.12	3.10 ± 0.12	0.90 ± 0.04	11.90 ± 0.49	9.30 ± 0.00	84.00 ± 0.58
RS-6	1.50 ± 0.03	2.80 ± 0.06	0.90 ± 0.04	10.87 ± 0.49	9.30 ± 0.17	0.00 ± 0.00
LSD	0.23	0.34	0.16	0.85	0.38	4.23

LSD (Fisher’s least significant differences); values are means ± standard error.

RS-1 (Floral head initiation): It is the initial stage of capitulum development. The average diameter of the capitulum was 3 mm in an angular diamond shape (Fig. 2a). In this stage, all the floral parts are green and indistinctly enclosed tightly in bracts. Floral head enlargement was a continuous process; it took three days to distinguish it to the next stage.

**Figure 3:**
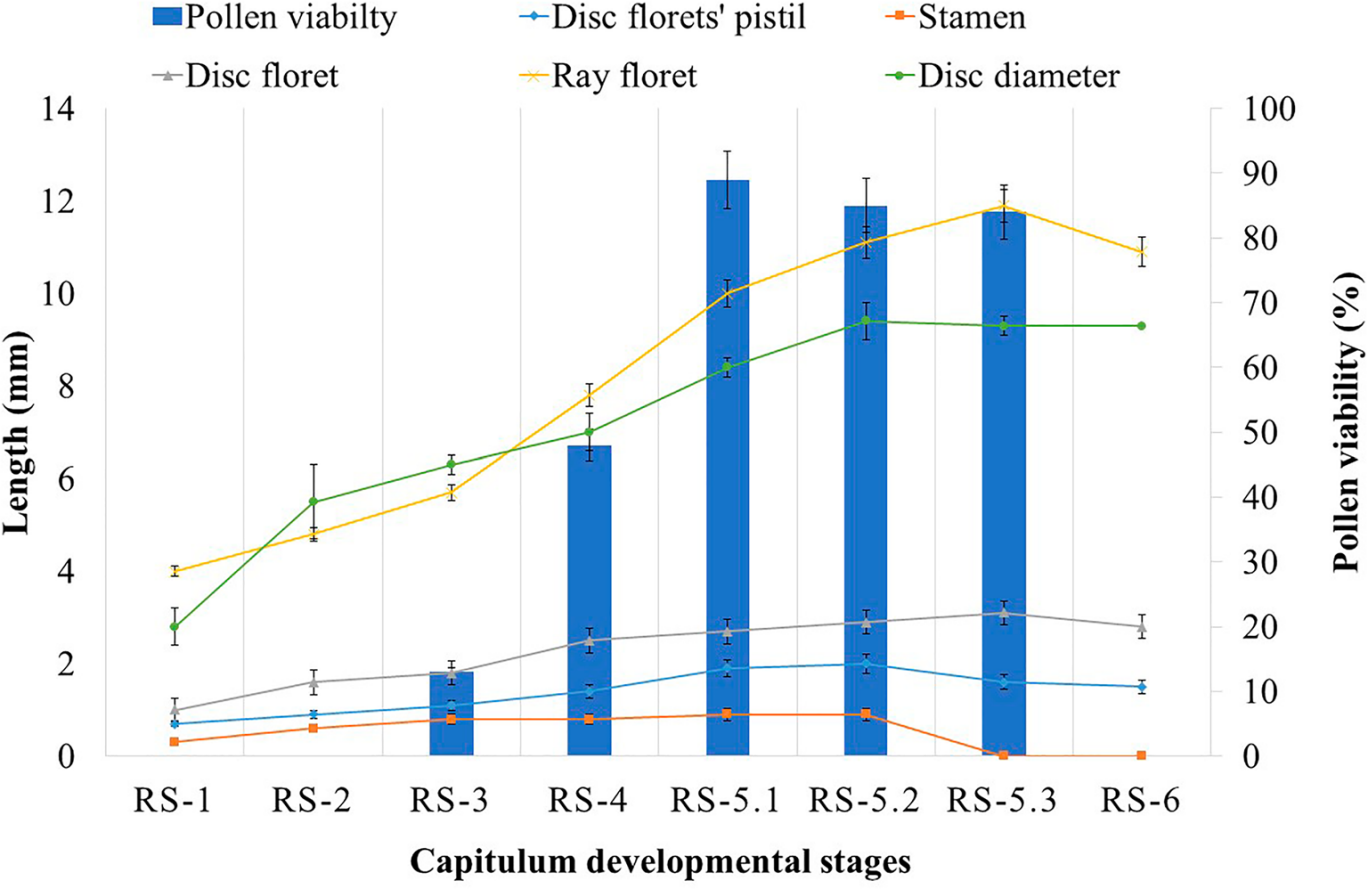
Variation in disc florets length, ray florets length, stamen length, disc florets' pistil length, capitulum diameter and pollen viability at various floral developmental stages.

RS-2 (Floral parts differentiation): The capitulum diameter increased to 5–6 mm, and white ray florets are distinctly visible from bracts. In this stage, stamens in the disc floret are yellowish-orange, while all remaining parts are yellowish-green in colour. The average length of ray florets (Fig. 2c), disc florets (Fig. 2b), disc florets’ pistils (Fig. 2d) and stamens (Fig. 2e) are 4.0, 1.2, 0.7 and 0.4 mm, respectively (Fig. 3). This stage lasts long up to two days.

RS-3 (Floral parts enlargement): Average diameter of the flower head in this stage was about 11 mm (Fig. 2a). Ray florets, pistils, and stamens attained lengths of 5.0, 1.0 and 0.5 mm (Fig. 2 and 3). Disc florets are of different lengths as per their location on the capitulum. The first whorl of disc florets towards ray florets was 1.7 mm, gradually decreasing for florets towards the tip of the capitulum. Ray florets are ready to open in this stage. Pollen production starts in the first whorl of disc florets during this stage, and light orange pollen grains are visible within anther sacs.

RS-4 (Anthesis starting point): This stage was identified as the starting point of anthesis. The ray floret was fully opened and about 8 mm in length (Fig. 2a,c and 3). The style branches of ray florets were ready to emerge. The first whorl of disc florets of length 2.5 mm was slightly swollen on its upper part and prepared for opening petals. Ovary of both the florets was also increased in length. Stamens of the first two to three whorl of disc florets are slightly increased in length and attained their maximum length (0.6 mm) in this stage. However, pollen viability was 48 percent at this stage. The average disc florets' pistil length was 1.5 mm in this stage. Both stages RS-3 and -4, took only one to two days to enter the florets in anthesis.

**Figure 4:**
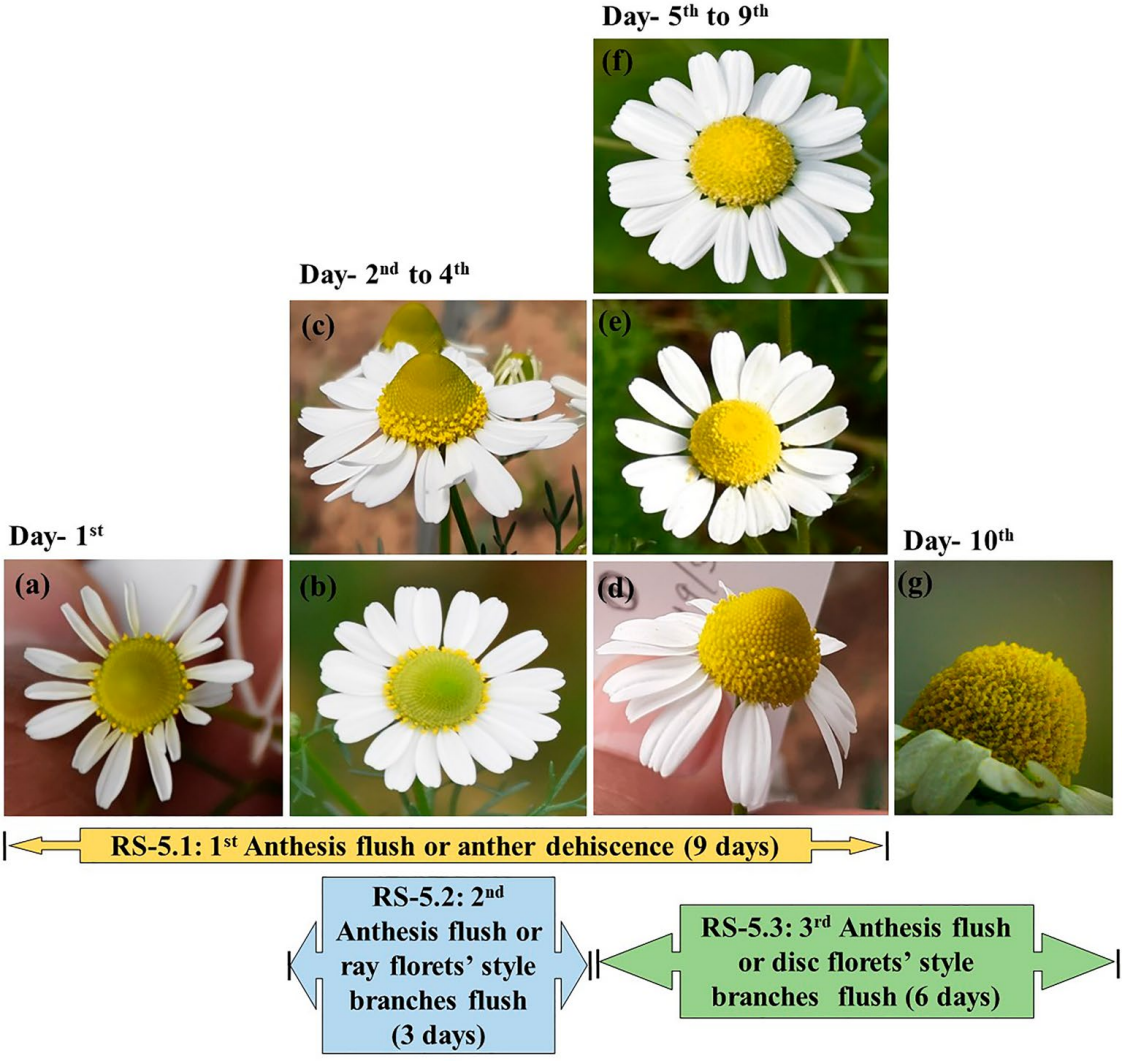
Sequential events of anthesis in German chamomile during reproductive stage (RS-5).

RS-5 (Anthesis flushes): This is the longest phase (10 days) and includes the reproductive events of the capitulum (Fig. 4). During this stage, the capitulum diameter was maximum (30 mm) (Fig. 3). This stage was further divided into three sub-stages according to the type and occurrence of anthesis flushes.

RS-5.1 (First anthesis flush or anther dehiscence flush): Opening first one or sometimes two whorls of disc florets and dehiscence of anthers was identified as first-ever flush in a capitulum. This flush starts six to seven days after primordium initiation and continues for the next nine days (Fig. 4a–f). It is the longest anthesis flush and overlaps with two style branches flushes (2nd and 3rd anthesis flushes) (Fig. 4b–f). Daily, two to three whorl of disc florets were opened, and anthers were dehiscence. The anthesis process of disc florets and the anther dehiscence occur following a centripetal order in the capitulum. The average length of disc florets at this time was 2.7 mm (Fig. 2b). At this stage, pollen viability was observed at its maximum (89 percent).

RS-5.2 (Second anthesis flush or ray florets' style branches flush): This flush of style branches emergence of ray florets occurs on the 2nd day after starting anther dehiscence and complete through all ray florets in two to three days (Fig. 4b–c). Ray florets' average length and style branches were 10 and 2 mm, respectively (Fig. 2c and d). Almost 40 percent of disc florets were opened and completed their anther dehiscence at the end of this style branches flush.

RS-5.3 (Third anthesis flush or disc florets, style branches flush): Style branches of disc florets started emerging almost on the 5th day of anthesis and continued for the next six days until the end of all anthesis flushes (Fig. 4d–g). This flush started when almost 50 percent of disc florets were completed anther dehiscence (Fig. 4d). The style branches of three or sometimes four whorls of disc florets emerged daily during this flush. The movement of style branches emergence generally took place in an upward direction towards the tip of the capitulum. Still, sometimes it was observed to start near the center and move in both directions. The average length of disc floret was at its maximum (3.3 mm) in this sub-stage (Fig. 2b).

RS-6 (Senescence and seed maturity): Ovary started turning light black at this stage, indicating initiation of seed development. Style branches showed senescence and turned brownish (Fig. 2d). Disc florets' corollas started drying but retained their dark yellow colour (Fig. 2b). Ray florets' corollas also retained their white colour but bent downward (Fig. 2a and c). The capitulum diameter was decreased due to the bending down of ray florets (Fig. 2a). At this stage, the average capitulum diameter was 12 mm. Seed matured in 15 to 17 days and dispersed automatically if not harvested before full maturity.

### Pollen viability and dispersal

**Figure 5:**
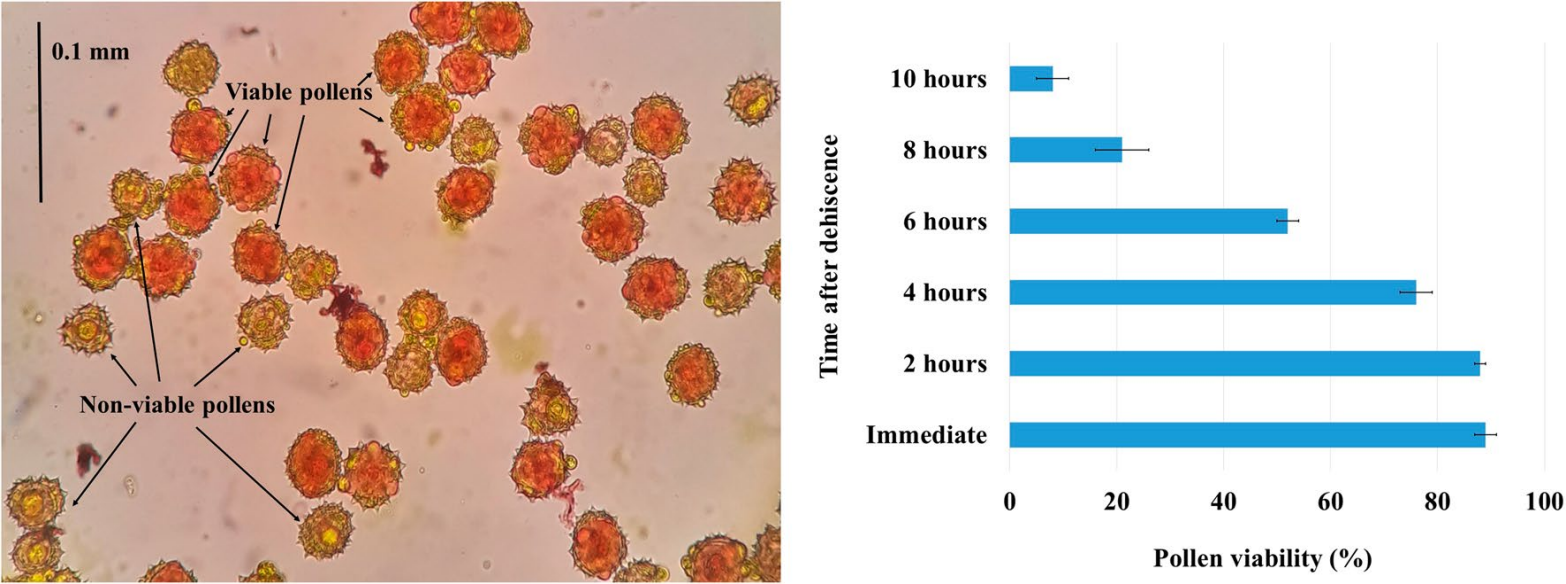
Pollen viability test at different time intervals after dehiscence.

Pollen viability was tested at different floral development stages and at different intervals after dehiscence. Maximum pollens were viable (89%) at the first anthesis flush (RS-5.1) stage (Table 2; Fig. 3). The pollen viability was at par for the first 2 h after anther dehiscence, but it was decreased further, and only 8 percent of pollens were found viable after 10 h (Fig. 5). Pollen viability was recorded 52 percent after 6 h of anther dehiscence. The decrease in pollen viability may be due to dehydration, temperature, humidity and other genetic factors of the species^39,40^. Several studies over the species showed a varied level of loss in pollen viability after anther dehiscence, which decides the time window for pollination either naturally or manually in a breeding program^41^. Furthermore, Pollens are sticky, complex spherical surfaces with tiny spikes in shape and, on an average of 0.032 mm in diameter (Fig. 5). Pollen showed dispersal through the air up to 0.7 m distance. However, high insect movement in the experiment plots showed that pollens were not limited to 0.7 m of air dispersal; despites were dispersed to several meters through insects.

### Breeding systems

**Figure 6:**
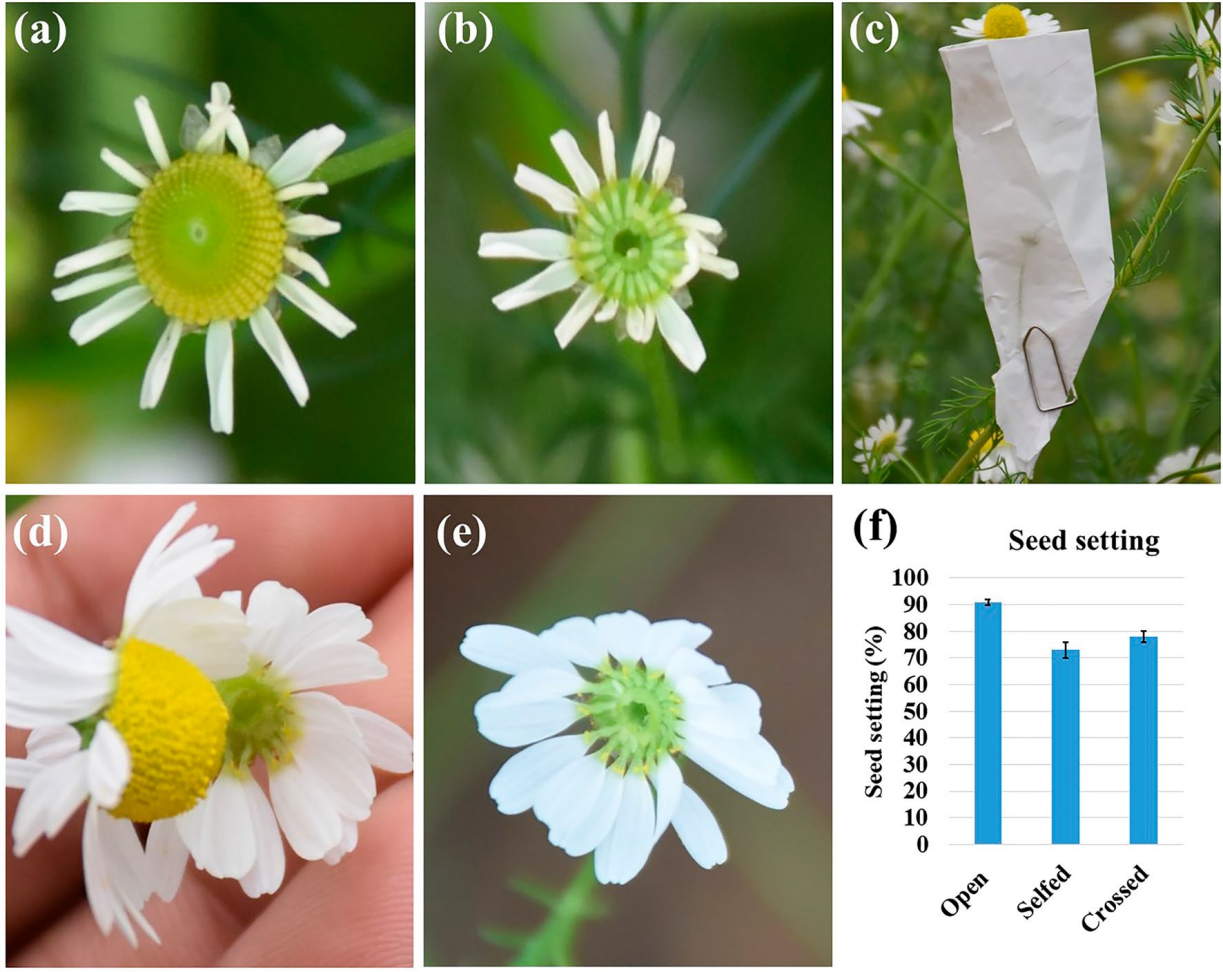
Controlled pollination experiments: (**a**) capitulum selected for selfing before anthesis (**b**) removing hermaphrodite disc florets, a practice mimic to emasculation (**c**) bagging for selfing/crossing (**d**) hand pollination (**e**) pollinated capitulum (**f**) seed setting percentage from all the controlled pollination experiments.

German chamomile could be believed cross-pollinated plant due to high insect movements during flowering^42^. However, the proportion of cross-pollination may vary over different varieties and ploidy levels. In the present study, analysis of variance for seed setting showed a highly significant (*p* ≤ 0.01) difference among all the three controlled pollination experiments (open pollination, selfed and crossed) (Fig. 6f). The results showed the highest (91%) viable seed setting in open pollination conditions (Fig. 6f). Few disc florets (3–9%) at the central tip of the capitulum were not developed into seeds, possibly due to tip sterility or improper development of florets. However, the seed set was also successfully achieved in selfed conditions (73%) and manually hand-pollinated experiments (78%), where all the disc florets were removed for controlled pollination. As the ray florets are the fertile pistillate florets, and stamens are present only in disc florets, disc florets removal was practiced for controlled pollination. The experiments showed that the practice of removal of disc florets could be successfully used as an alternate to emasculation in German chamomile (Fig. 6a–e). Moreover, comparable seed settings in selfed conditions indicated the possibility of often-cross pollination behavior in this plant.

### Essential oil content and compositions at different floral development stages

**Figure 7:**
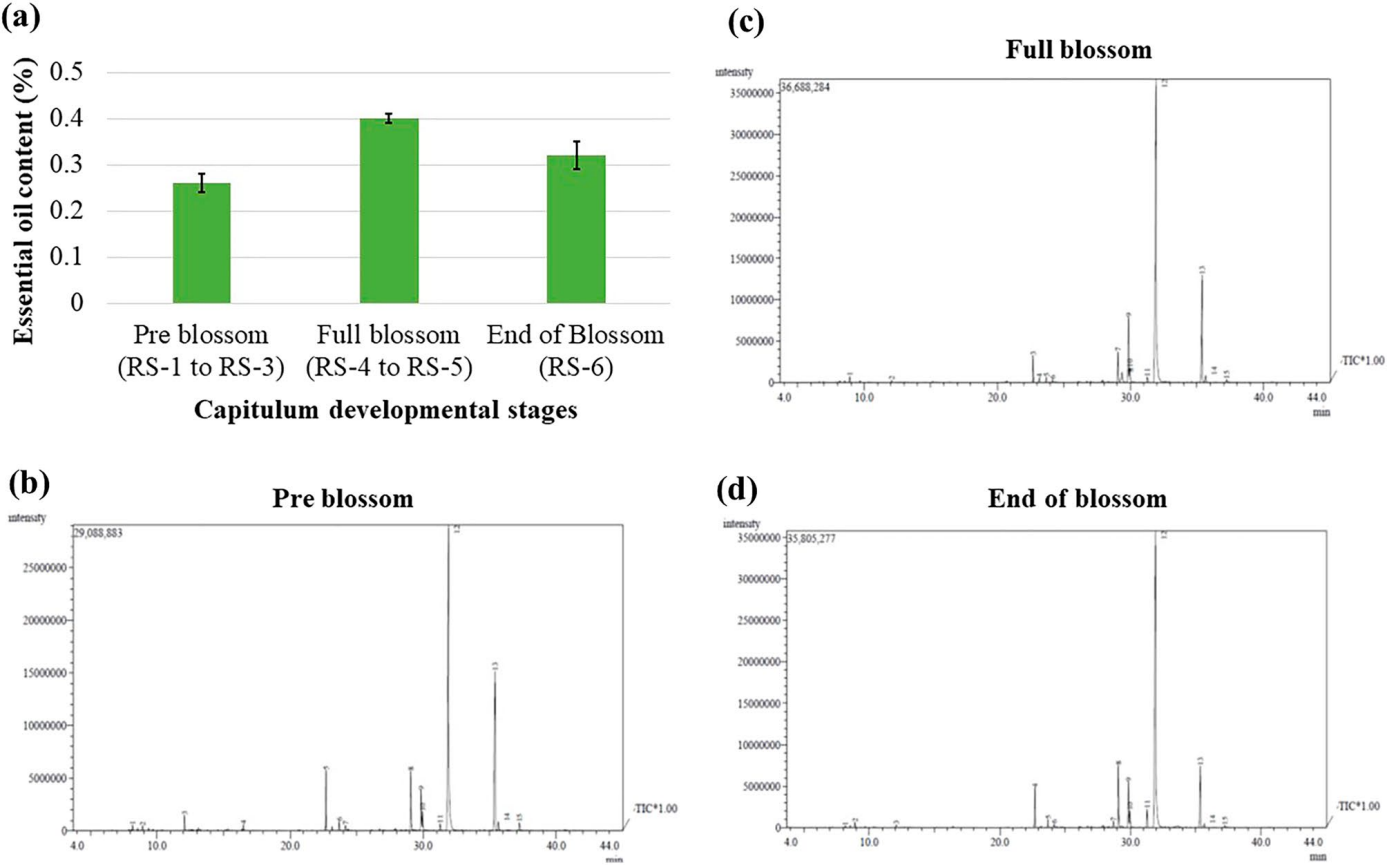
Essential oil content (**a**) and its chromatograms (**b**–**d**) at pre blossom (RS-1 to RS-3), full blossom (RS-4 and -5), and end of blossom (RS-6) in German chamomile.

In the present study, the blue essential oil content was recorded highest 0.40% at full blossom stage (RS-4 & -5) of capitulum development, followed by 0.32% at the end of blossom stage (RS-6) (Fig. 7a). Essential oil extraction from fresh capitula and a diploid population might be the reasons for the lower essential oil percentage compared to earlier studies^12,13^. The quality of blue essential oil depends on chamazulene and bisabolol oxide-A content^6^. The essential oil extracted from German chamomile is blue due to chamazulene content^43^, produced by matricine breakdown during heat distillation. Overall, seventeen compounds were detected in the essential oil from all the three floral developmental stages, *i.e.*, pre-blossom (Fig. 7b), full blossom (Fig. 7c) and end of blossom (Fig. 7d) by GC–MS analysis. The chamazulene formation during steam distillation was found highest (2.419%) at end of blossom stage (RS-6) of capitulum development followed by full blossom (RS-4 & -5) (Table 3), which might be genetically controlled towards pathways for developmental stages^44^. Likewise, α-bisabolol and bisabolol oxide-A was also higher (1.920% and 65.100%, respectively) during end of blossom stage (RS-6) followed by full blossom (RS-4 & -5) (Table 3). The higher bisabolol oxide-A (65.100%) was may be due to high receptivity of bisabolol to oxidation and converted to bisabolol oxide-A^45^.

**Table 3:** Chemical constituents of blue essential oil during different floral development stages (pre blossom, RS-1 to RS-3; full blossom, RS-4 and -5; end of blossom, RS-6) in German chamomile.

Sr. no	Compound Name	Retention time	Retention index	Pre-blossom (%)	Full blossom (%)	End of blossom (%)	Least significant difference
1	Eucalyptol(1,8-Cineole)	8.167	1036	0.44 ± 0.23	–	0.24 ±	–
2	Artemisia ketone	8.918	1059	0.32 ± 0.01	0.48 ± 0.01	0.48 ± 0.01	0.02
3	Camphor	12.068	1150	1.42 ± 0.01	0.18 ± 0.01	0.29 ± 0.01	0.02
4	4-Undecanone	16.493	1272	0.44 ± –	–	–	–
5	Beta-Farnesene	22.693	1452	5.51 ± 0.80	2.68 ± 0.39	4.28 ± 0.63	0.26
6	Germacrene D	23.689	1481	0.75 ± 0.10	0.53 ± 0.01	0.89 ± 0.04	0.13
7	Bicyclogermacrene	24.162	1495	0.36 ± 0.00	0.29 ± 0.00	0.40 ± 0.00	0.01
8	Cadinol < epi-α- >	28.704	1643	–	–	0.75 ± –	–
9	Bisabolol oxide B < α- >	29.071	1655	6.24 ± 1.00	3.53 ± 0.33	–	1.14
10	Phloroacetophenone < 2,4-dimethylether- >	29.371	1665	–	1.67 ± 0.01	–	–
11	Bisabolone oxide A < α- >	29.848	1681	3.95 ± 0.59	7.67 ± 1.01	5.78 ± 1.03	1.33
12	Bisabolol < α- >	29.952	1684	1.77 ± 0.00	1.46 ± 0.00	1.92 ± 0.09	0.04
13	Chamazulene	31.271	1733	0.69 ± 0.00	0.64 ± 0.06	2.42 ± 0.09	0.18
14	Bisabolol oxide A	31.897	1757	51.51 ± 3.00	62.32 ± 0.83	65.1 ± 2.13	3.79
15	En-in-dicycloether	35.406	1882	25.03 ± 0.72	17.00 ± 1.62	8.73 ± 1.00	2.12
16	En-in-dicycloether	35.655	1890	0.85 ± 0.00	0.81 ± 0.06	0.46 ± 0.00	0.04
17	1,4-Naphthalenedione, 2-hydroxy-3-(1-propenyl)	37.230	1942	0.71 ± 0.01	0.30 ± 0.00	0.21 ± 0.01	0.05

The above results are in accordance of earlier reports showed that essential oil content in German chamomile largely depends on the stage of capitulum development^14,15^. Several biosynthesis pathways may be disturbed and remained immature during differential harvesting^21,22^, which might be the reason for the variation in quality as well as quantity of essential oil harvested at different floral developmental stages. Beside this, there are several non-genetic factors like time of harvesting^46^, nutrient doses^1,47^, the gap between harvesting and distillation^27,28^ and abiotic factors under mega-environments^8,9^, which may affect the quality and quantity of blue essential oil. The observations of present study indicated that the highest quality oil was extracted from the end of blossom stage. The essential oil content and composition findings at different capitulum development stages suggested that the capitulum picking should be done from anthesis onwards when most of the capitula are at the end of anthesis (RS-5.3) or post anthesis (RS-6). Salehi and Hazrati^48^; and Kumar et al^46^ reported that the quality and quantity of essential oil were higher at noon, which may be because during noon hours' capitula are at full blossom and in the anthesis phase. Similarly, Kumar et al.^23^ suggested post anthesis as the right stage of harvesting wild marigold for higher essential oil content and quality. The percent distribution of constituents and principal component analysis over three floral developmental stages also depicted that full blossom to end of blossom stages contributed the maximum variability in chamazulene and bisabolol contents in German chamomile.

**Figure 8:**
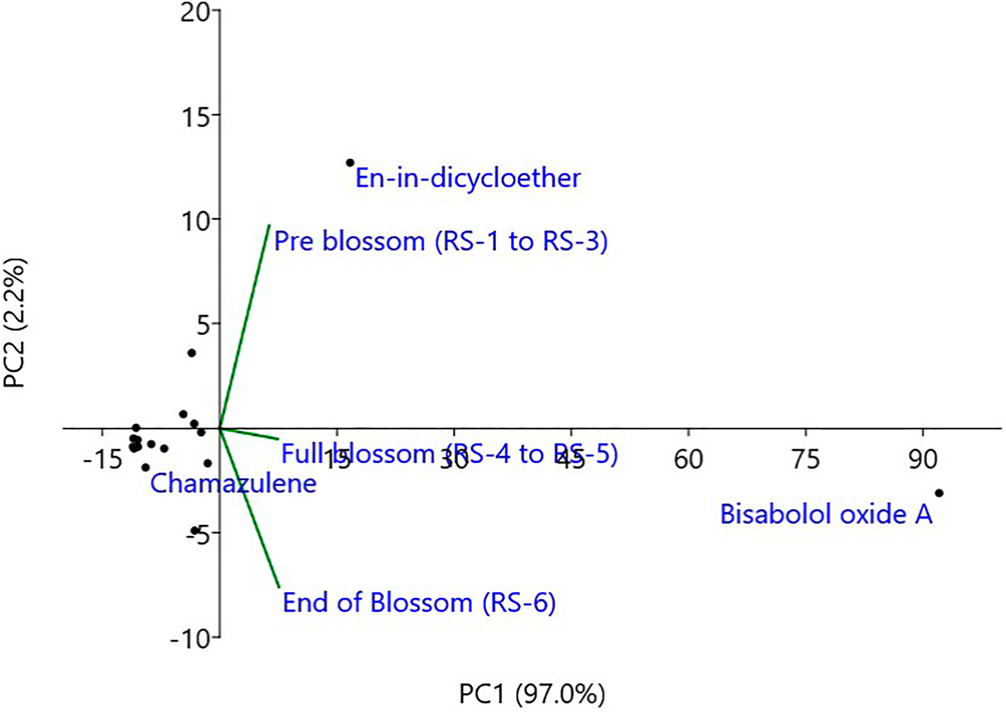
Principal components analysis of all the essential oil constituents at pre blossom (RS-1 to RS-3), full blossom (RS-4 and -5), and end of blossom (RS-6) in German chamomile.

The first two principal components (PCs) were contributed 99.2% (PC1-97.0% and PC2-2.2%) to the total variation (Fig. 8). The Eigenvector for the full blossom stage was very close to PC1 in a positive direction, showing that this stage contributed highest and positively to the total variation among chemical components. Also, the 'bisabolol oxide A' component was on the positive extreme of PC1, indicating that this component was changed to the maximum variation towards full blossom stages. However, the full blossom and end of blossom stages had negative values on PC2, which showed that the major positive contributor for PC2 (en-in-dicycloether) was decreasing towards the end of blossom. The variable constituents over capitulum development showed that the stage of capitula during picking is crucial for quality aspects of blue essential oil. Therefore, future research on the quality of blue essential oil should consider the effects of capitulum development stages. For the farmers and commercial growers, the right stage of capitula harvest is when the capitulum is at full blossom or towards the end of the blossom. Overall, these findings suggested the optimal stage of capitula picking to get maximum quality and quantity of blue essential oil.

## Conclusions

The present study investigated several aspects of breeding systems, reproductive biology, floral development, essential oil content and composition during floral developmental stages. The species has pistillate ray florets and perfect disc florets on the capitulum, which is a unique character of the Asteraceae family. The complete floral development could be divided into six reproductive phases. The fifth stage of floral development (RS-5) continued for ten days, involving three anthesis flushes: anther dehiscence, ray florets' style branches and disc florets' style branches flush. Removal of disc florets manually with forceps could be successfully used as an alternate option for emasculation in German chamomile. Moreover, chemical insights during floral developmental stages revealed that the full blossom stage (RS-4 & -5) has maximum essential oil content (0.40%), while the end of blossom stage (RS-6) has given the highest bisabolol oxide-A (65.100%) and chamazulene content (2.4192%). The results on essential oil content and constituents variation suggested the optimal stage of capitula picking to get maximum quality and quantity of blue essential oil. Besides the growth period and optimum harvesting stage in Himalayan mid-hill conditions, the present study mainly suggested an alternate emasculation technique of crossing and sequential happenings of all the anthesis flushes, which would have practical implications in selfing and crossing practices in German chamomile. Also, the study would be the base for future experiments on the generation of new variations using hybridization, breeding line development programs, improvement of quality essential oil and optimal mechanical harvesting. Overall, the present study's results on reproductive biology and breeding systems would open several aspects related to tools and techniques for plant breeders.

## Data Availability

The data that support the findings of this study are available within the tables and figures of the manuscript.
